# Dismal prognosis of patients with operative indication without surgical intervention in active left-sided infective endocarditis

**DOI:** 10.3389/fcvm.2023.1223878

**Published:** 2023-08-24

**Authors:** Mathias Van Hemelrijck, Juri Sromicki, Michelle Frank, Matthias Greutmann, Bruno Ledergerber, Jana Epprecht, Maria Padrutt, Paul R. Vogt, Thierry P. Carrel, Omer Dzemali, Carlos-A. Mestres, Barbara Hasse

**Affiliations:** ^1^ Department of Cardiac Surgery, University Hospital Zurich, Zurich, Switzerland; ^2^ Department of Cardiology, University Hospital Zurich, Zurich, Switzerland; ^3^ Department of Infectious Diseases and Hospital Epidemiology, University Hospital Zurich, Zurich, Switzerland

**Keywords:** infective endocarditis, cardiac surgery, operative indication, endocarditis team, antimicrobial treatment

## Abstract

**Introduction:**

Around 25% of patients with left-sided infective endocarditis and operative indication do not undergo surgery. Baseline characteristics and outcomes are underreported. This study describes characteristics and outcomes of surgical candidates with surgical intervention or medical treatment only.

**Methods:**

Retrospective analysis of ongoing collected data from a single-center from an observational cohort of patients with infective endocarditis (ENVALVE). Kaplan-Meier estimates for survival was calculated. Factors associated with survival were assessed using a bivariable Cox model. To adjust for confounding by indication, uni- and multivariable logistic regression for the propensity to receive surgery were adjusted.

**Results:**

From January 2018 and December 2021, 154 patients were analyzed: 116 underwent surgery and 38 received medical treatment only. Surgical candidates without surgery were older (70 vs. 62 years, *p *= 0.001). They had higher preoperative risk profile (EuroSCORE II 14% (7.2–28.6) vs. 5.8% (2.5–20.3), *p *= 0.002) and more comorbidities. One patient was lost-to-follow-up. Survival analysis revealed a significant higher one-year survival rate among patients following surgery (83.7% vs. 15.3% in the non-surgical group; log-rank test <0.0001). In the final multivariable adjusted model, surgery was less likely among patients with liver cirrhosis [OR = 0.03 (95% CI 0.00–0.30)] and with hemodialysis [OR = 0.014 (95% CI 0.00–0.47)].

**Conclusion:**

Patients with left-sided infective endocarditis who do not undergo surgery despite an operative indication are older, have more comorbidities and therefore higher preoperative risk profile and a low 1-year survival. The role of the Endocarditis Team may be particularly important for the decision-making process in this specific group.

## Introduction

Infective endocarditis (IE) is a complex disease requiring multidisciplinary management as recommended in current Clinical Practice Guidelines (1). In addition to antimicrobial therapy, 25%–50% of the patients will undergo surgery during the active phase of the disease (2), but overall mortality remains high, ranging from 10%–30% (3–5). Advantages of operative intervention are well established (4, 6, 7): ability to remove infected tissue, to prevent future embolization, stroke, heart failure and thereby to reduce morbidity and mortality (4, 5, 8). Despite these potential advantages, approximatively 25% of patients with a clear-cut operative indication will receive medical treatment only (9–13). Reasons to deny surgery are multiple, making it difficult to establish universal recommendations. The decision-making process including surgical indication, patient profile, patient and relatives wishes, but also the potential benefit on the expected life expectancy is not yet well understood. The aim of this study was to evaluate characteristics, in-hospital and one-year mortality among patients with active left-sided infective endocarditis (ALSIE) who all clearly fulfilled criteria for a surgical indication and that received surgical intervention or medical treatment only.

## Methods

### Study design and data collection

This is a retrospective analysis of prospectively collected data from an observational endocarditis cohort ENVALVE (ENdovascular and cardiac VALVE infection Cohort) in a tertiary center (University Hospital Zurich—USZ) in Switzerland. Data collection forms containing demographic, clinical, laboratory, and treatment information are completed by physicians and study nurses since January 2018. Decision on operability or rejection is made by the USZ Endocarditis Team according to the most actual Clinical Practice Guidelines and is based on patients' characteristics at the time of presentation (Modified Duke criteria, age, clinical condition, neurological status, likelihood of survival with surgery, quality of life) (1, 12) and patients' wish.

### Study participants

ENVALVE participants aged 18 or older with ALSIE of a native or a prosthetic valve and with confirmed indication for surgery according to current Clinical Practice Guidelines (1) and at least two cohort visits from 1 January 2018 through 31 December 2021 were included. Patients were stratified in two groups for further comparison: **group 1)** operative indication and subsequent surgery, and **group 2)** operative indication but no surgery performed. We excluded patients with isolated right-sided IE, intracardiac device-related IE and aortic graft infection from the analysis.

### Definitions

The modified Duke Criteria and the proposed modification from the European Society of Cardiology in 2015 were used for the diagnosis of IE since it has been shown that these criteria have a high sensitivity (1, 14). Timing for surgery was divided in three different scenarios: elective (defined as surgery after 1–2 weeks of antimicrobial therapy); urgent (within a few days, <7 days); or emergent (within 24 h) irrespective of antimicrobial treatment duration (1). Operative mortality was defined according to the *Society of Thoracic Surgeons* (15)*.* Charlson comorbidity index (CCI) (16), EuroSCORE II (17) and the APORTEI predicted mortality risk score (18) were calculated to estimate perioperative morbidity and mortality. The latter has been lately validated as a similar risk stratification score for IE (18). Perioperative complications were reported as per guidelines for reporting mortality and morbidity after cardiac valve interventions (19).

Infection with hepatitis-B-Virus (HBV) was confirmed in patients with positive hepatitis-surface antigen and positive HBV DNA in laboratory testing, or in those under medical treatment. Infection with hepatitis C-Virus (HCV) was confirmed in patients with positive serological testing and detectable HCV RNA or in those under medical treatment. The Child-Pugh Score was calculated for the preoperative assessment of cirrhosis mortality.

### Statistical analysis

Statistical analyses were conducted using Stata 17.0. Qualitative variables were expressed as numbers and percentages. Quantitative variables were expressed as median and interquartile range (IQR). For qualitative variables Fisher's exact test and for continuous variables the Mann-Whitney-U test were used, whenever applicable. Statistical significance was achieved when *p *< 0.05. Kaplan-Meier estimates were calculated for survival and are depicted with 95% confidence intervals. Log rank pairwise comparisons were performed to determine different distributions.

We assessed factors associated with survival including surgery using a bivariable Cox model. Many risk factors influence both the decision to undergo surgery and survival. To adjust for this confounding by indication we performed a multivariable logistic regression to summarize the probability of receiving surgery in a propensity score including age, sex, CCI, diabetes mellitus, hepatitis B, liver cirrhosis, cerebrovascular disease, dialysis, estimated glomerular filtration rate (eGFR) at diagnosis, creatinine at diagnosis, malignancy, ischemic stroke, haemorrhagic stroke, *Staphylococcus aureus*, *Streptococcus* spp, intubation, cardiogenic shock, septic shock, EuroSCORE II and APORTEI score. The score was then included as an interaction term in the Cox models for surgery, used both as untransformed linear variable as well as after stratification into quintiles (20).

### Ethics

The Ethics Committee/Institutional Review Board approved the study within the framework of the ENVALVE-cohort (BASEC 2017-01140). Written informed consent was obtained from all participants.

## Results

### Patient population

Between January 2018 and December 2021, we identified 146 patients with definitive ALSIE and 8 patients with possible ALSIE and a confirmed operative indication. Out of them, 70% had indication to surgery because of hemodynamic deterioration, 45% because uncontrolled infection and 25% to prevention embolization (more than one indication possible). Overall, 116 patients were operated (**group 1**), and 38 were denied for surgery and received medical treatment only (**group 2**).

The timing of surgery of the 116 patients who were operated was elective (72%), emergent (22%) and urgent (6%), and the median interval from diagnosis to surgery was 16 days [interquartile range (IQR) 8.5–28.5]. Table 1 shows the prevalence of native-valve endocarditis (NVE) and prosthetic valve endocarditis (PVE) as well as baseline characteristics. Differences between the two groups were found in age [62 years (IQR 48–70) vs. 70 years], diabetes mellitus (13 vs. 29%), eGFR (75 ml/min (50–97) vs. 41 ml/min (33–84)), dialysis (1% vs. 9%), positive hepatitis B virus (HBV) status (2 vs. 13%), and end-stage liver disease. Furthermore, differences were observed in CCI (2 units (1–5) vs. 6 units (3–9), EuroSCORE II (6% (3–21) vs. 13% (7–29)) and APORTEI mortality scores (12% (8–25.7) vs. 33% (22–52). There were also differences in hemorrhagic stroke (8 vs. 21%), renovisceral embolism (24 vs. 42%), intubation (9 vs. 26%), cardiogenic shock (11 vs. 26%) and septic shock (9 vs. 29%).

**Table 1:** Baseline characteristics of 154 patients with left sided infective endocarditis with surgical indication.

Baseline characteristics	Operated patients	Not-operated patients	*P*-value
	*n *= 116	*n *= 38	
Type of endocarditis			
NVE, *n* (%)	96 (83)	27 (71)	0.160
PVE, *n* (%)	21 (18)	11 (29)	0.170
Aortic valve, *n* (%)	61 (52)	16 (42)	0.350
Mitral valve, *n* (%)	73 (63)	26 (68)	0.566
Tricuspid valve, *n* (%)	2 (1)	0 (0)	1
Risk factors			
Age, median years (IQR)	62 (48–70)	70 (62–79)	< 0.001
Male, *n* (%)	88 (76)	25 (66)	0.290
BMI, median kg/m^2^ (IQR)	25 (22–28)	25 (22–31)	0.900
Charlson comorbidity index, median points (IQR)	2 (1–5)	6 (3–9)	<0.001
Diabetes mellitus, *n* (%)	15 (13)	11 (29)	0.043
Arterial hypertension, *n* (%)	54 (47)	22 (58)	0.264
CAD, *n* (%)	29 (25)	10 (26)	1
PVD, *n* (%)	6 (5)	4 (10)	0.264
CVD, *n* (%)	10 (8)	8 (21)	0.076
Previous stroke, *n* (%)	13 (11)	7 (18)	0.271
COPD, *n* (%)	8 (7)	6 (16)	0.111
eGFR, median ml/min (IQR)	75 (50–97)	41 (33–84)	< 0.001
Dialysis, *n* (%)	1 (1)	8 (21)	< 0.001
Malignancy, *n* (%)	19 (16)	12 (31)	0.061
HBV, *n* (%)	3 (2)	5 (13)	0.023
HCV, *n* (%)	7 (6)	3 (8)	0.709
Liver cirrhosis, Child-Pugh A, *n* (%)	1 (1)	3 (8)	0.047
Liver cirrhosis, Child-Pugh B, *n* (%)	1 (1)	3 (8)	0.047
Liver cirrhosis, Child-Pugh C, *n* (%)	0 (0)	3 (8)	0.014
Previous cardiac surgery, *n* (%)	22 (19)	13 (34)	0.073
LVEF, median % (IQR)	60 (55–65)	58 (50–61)	0.092
NYHA III/IV, *n* (%)	57 (49)	21 (55)	0.577
HIV, *n* (%)	1 (1)	1 (2)	0.434
Surgery			
EuroSCORE II, median % (IQR)	6 (3–21)	13 (7–29)	0.014
APORTEI mortality, median % (IQR)	12 (8–25.7)	33 (22–52)	<0.001
Microbiology			
Positive blood cultures, *n* (%)	105 (90)	37 (97)	0.296
*Staphylococcus aureus*, *n* (%)	33 (28)	20 (52)	0.010
Coagulase-negative staphylococci, *n* (%)	7 (6)	5 (13)	0.171
*Streptococci* spp, *n* (%)	42 (36)	6 (18)	0.025
*Enterococci* spp, *n* (%)	13 (11)	4 (10)	1
Negative-cultures, *n* (%)	5 (3)	0 (0)	0.237
Negative-cultures, positive PCR, *n* (%)	6 (5)	0 (0)	0.337
Complications			
Ischemic stroke, *n* (%)	48 (41)	10 (26)	0.123
Hemorrhagic stroke, *n* (%)	9 (8)	8 (21)	0.035
Renovisceral embolism, *n* (%)	28 (24)	16 (42)	0.040
Intubation, *n* (%)	11 (9)	10 (26)	0.014
Cardiogenic shock, *n* (%)	13 (11)	10 (26)	0.034
Septic shock, *n* (%)	11 (9)	15 (39)	<0.001

NVE, native valve endocarditis; PVE, prosthetic valve endocarditis*;* BMI, body mass index in kg/m^2^; CAD, coronary artery disease; PVD, peripheral artery disease; CVD, cerebrovascular disease; COPD, chronic obstructive pulmonary disease; GFR, glomerular filtration rate in ml/min; HBV, hepatitis B virus; HCV, hepatitis C virus; HIV, human immunodeficiency virus. LVEF, left-ventricular ejection fraction; PCR, polymerase chain reaction; NYHA, New York Heart Association.

Reasons to deny surgery are shown in Table 2. There was more than one reason for surgical denial in 13 (34%) patients. The most common cause was an expected high perioperative mortality (30% of patients).

**Table 2:** Reason for denied surgery in 38 patients.

Reason for no surgery	*n* (%)
Stroke	1 (2)
Ongoing IVDU	2 (5)
Patients’ wish	4 (10)
Age	6 (16)
Multimorbidity	6 (16)
Malignancy	6 (16)
Intracranial bleeding	8 (21)
End-stage liver disease	9 (23)
High expected mortality	11 (30)

IVDU, intravenous drug use.

One or more reasons are possible. The addition of percentages exceeds 100%.

### Follow-up

**Figure 1:**
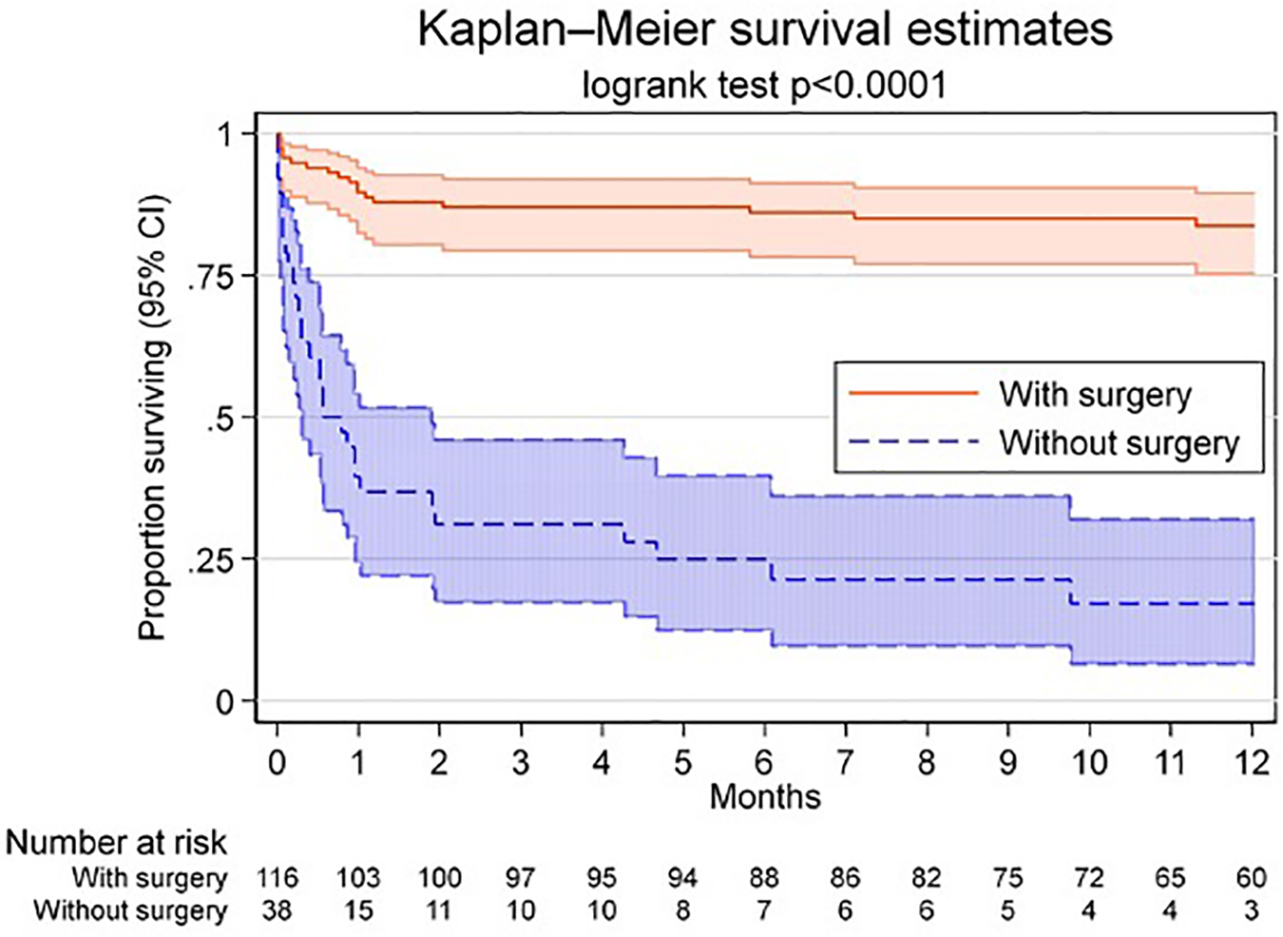
Kaplan-Meier estimates for survival with 95% CI (shadowing). Kaplan-Meier survival analysis for patients with operative indication with and without surgical intervention. Shadowing shows 95% confidence intervals (CI).

Median follow-up was 283 days (IQR 32–455). Figure 1 depicts survival estimates at follow-up. At 1 year, 84% (CI 95% 77–91) in group 1 and 17% (CI 95%, 3–30) in group 2 were alive (log-rank test <0.0001).

### Uni- and bivariable analysis

**Figure 2:**
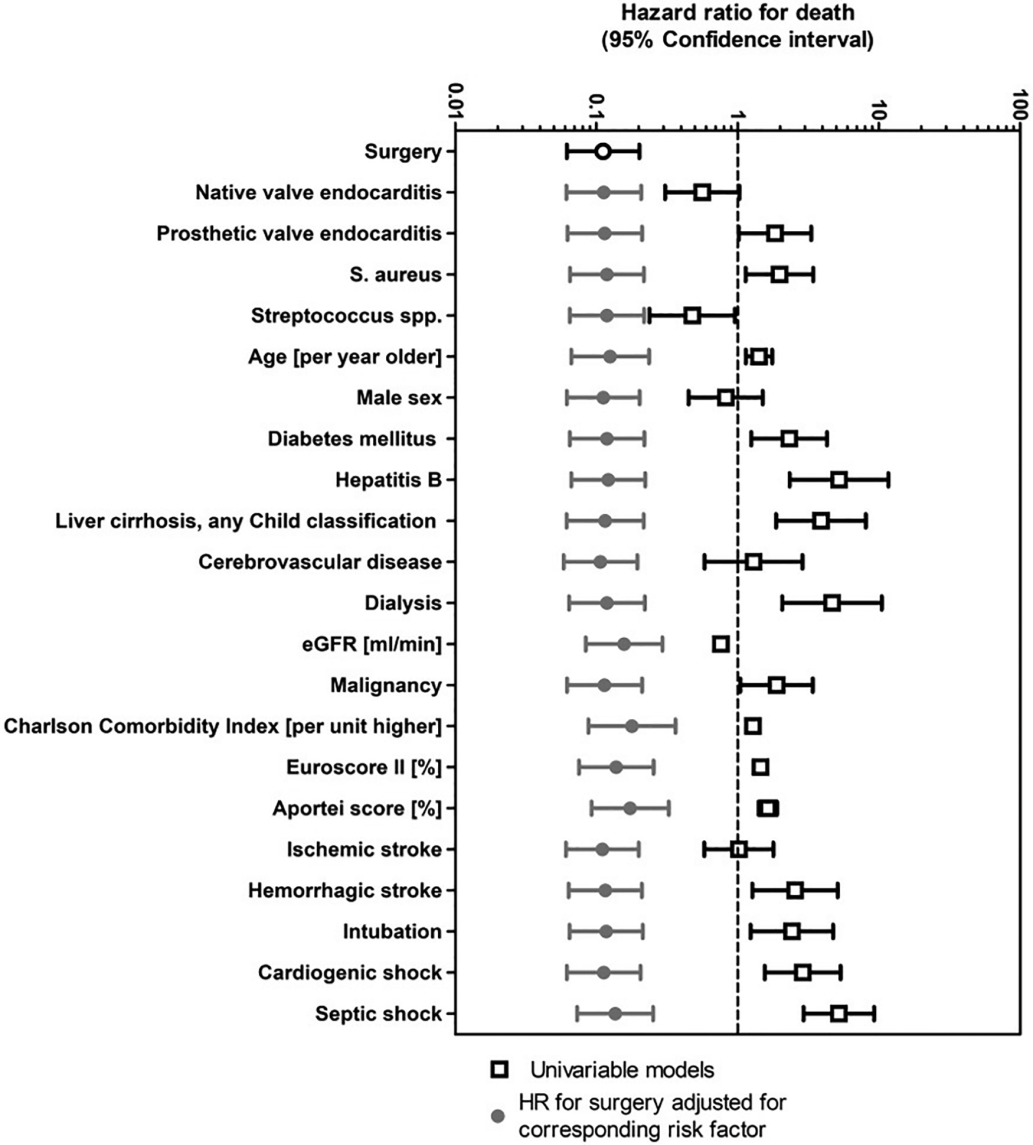
Uni- and bivariable Cox regression for death of 154 patients with left sided infective endocarditis and operative indication. We present univariable associations of death. Many risk factors influence both the decision to undergo surgery and also death. We therefore show bivariable associations with surgery adjusted for all parameters listed. eGFR, estimated glomerular filtration rate; HR, hazard ratio; S. aureus, staphylococcus aureus.

Univariable analyses showed that surgery had a very strong positive effect on death prevention (HR 0.11, 95% CI 0.062–0.202, *p* < 0.001). We also examined the effect of surgery in bivariable Cox models and all risk factors without finding relevant modifications (Figure 2). The point estimates for the hazard ratios based on the propensity score and the bivariable analysis with different additional variables varied between 0.11 and 0.18 for the endpoint surgery.

### Uni- and multivariable logistic regression and adjusted analyses for the propensity to receive surgery

**Figure 3:**
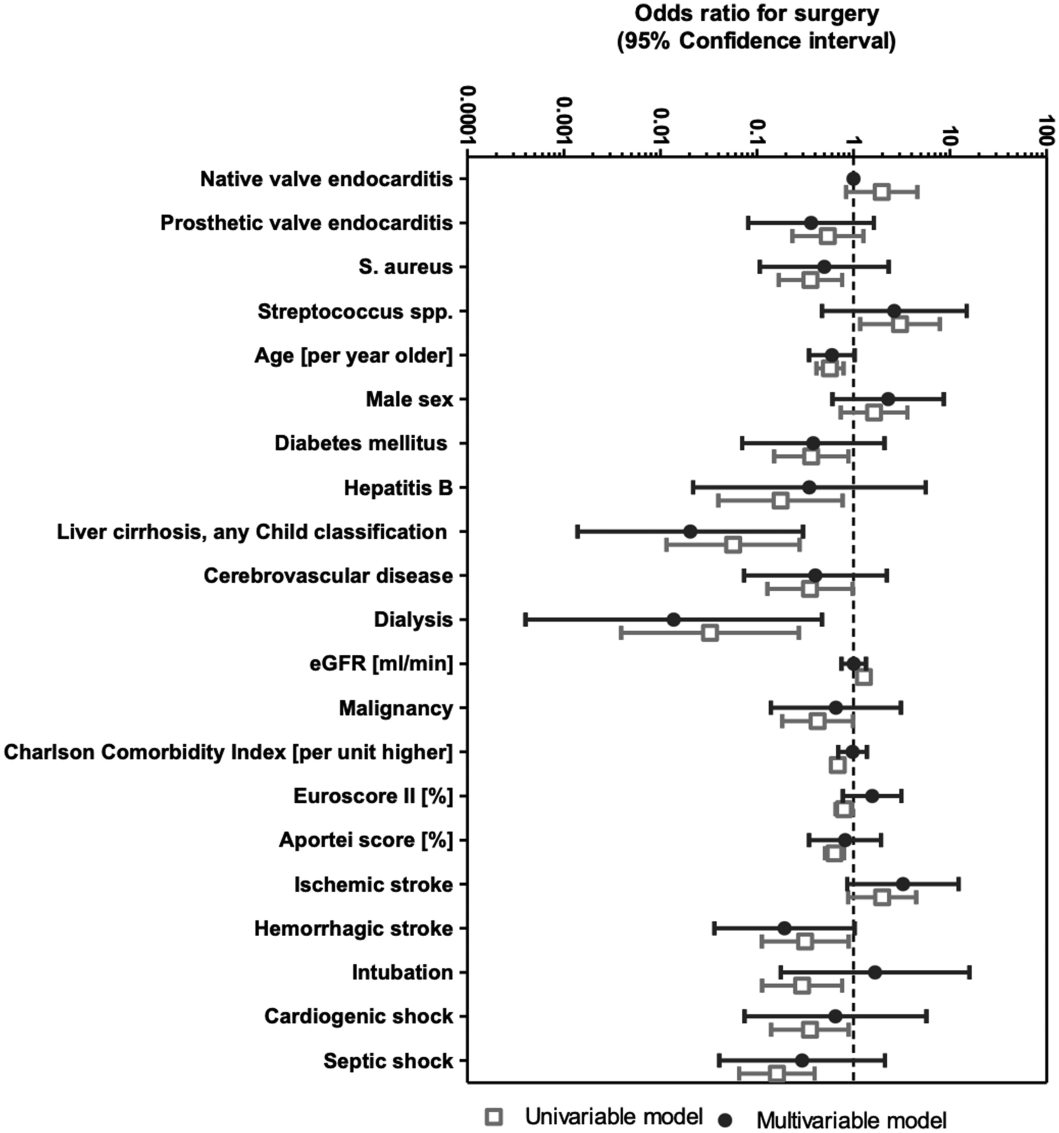
Multivariable logistic regression for the propensity to receive surgery for infective endocarditis. Multivariable models were adjusted for all parameters listed. eGFR, estimated glomerular filtration rate; S. aureus, staphylococcus aureus.

Uni- and multivariable odds ratios (OR) for surgery are shown in Figure 3. Univariable logistic regression showed associations for surgery with streptococcal IE [OR 3.03 (95% CI 1.2–7.8)] and eGFR at diagnosis [OR 1.3 (95% CI 1.1–1.5)]. There was also a trend for a positive association with ischemic stroke [OR 1.98 (95% CI 0.88–4.45)]. There was evidence of negative association of surgery with age [OR 0.57 (95% CI 0.41–0.79)], diabetes mellitus [OR 0.36 (95% CI 0.15–0.88)], hepatitis B [OR 0.18 (95% CI 0.04–0.77)], liver cirrhosis [OR 0.06 (95% CI 0.01–0.28)], cerebrovascular disease [OR 0.35 (95% CI 0.13–0.98)], dialysis [OR 0.03 (95% CI 0.003–0.27)], malignancy [OR 0.42 (95% CI 0.18–0.99)], CCI [OR 0.35 (95% CI 0.14–0.89)] and *S. aureus* [OR 0.36 (95% CI 0.17–0.76)]. Surgery was less likely among patients with high surgical risk scores (EuroSCORE II [OR 0.78 (95% CI 0.65–0.97)]; APORTEI [OR 0.63 (95% CI 0.51–0.79)], and also among patients with complications due to IE such as hemorrhagic stroke [OR 0.32 (95% CI 0.11–0.89)], necessity for intubation and ventilation [OR 0.29 (95% CI 0.11–0.76)], cardiogenic shock [OR 0.35 (95% CI 0.14–0.89)] and septic shock [OR 0.32 (95% CI 0.07–0.39)].

In the final multivariable adjusted model, surgery was less likely among patients with liver cirrhosis [OR = 0.03 (95% CI 0.00–0.30)] and with dialysis [OR = 0.014 (95% CI 0.00–0.47)]. There was also trend to less frequent surgery in patients with ischemic (OR 3.24 [95% CI 0.86–12] and hemorrhagic stroke (OR 0.19 [95% CI 0.04–1.0]. Because of correlations between several of these variables, it is difficult to judge the contribution of individual components in a multivariable model adjusted for all variables. However, this multivariable model allowed for a very good prediction of individual propensity scores to receive surgery (ROC AUC = 0.92).

**Figure 4:**
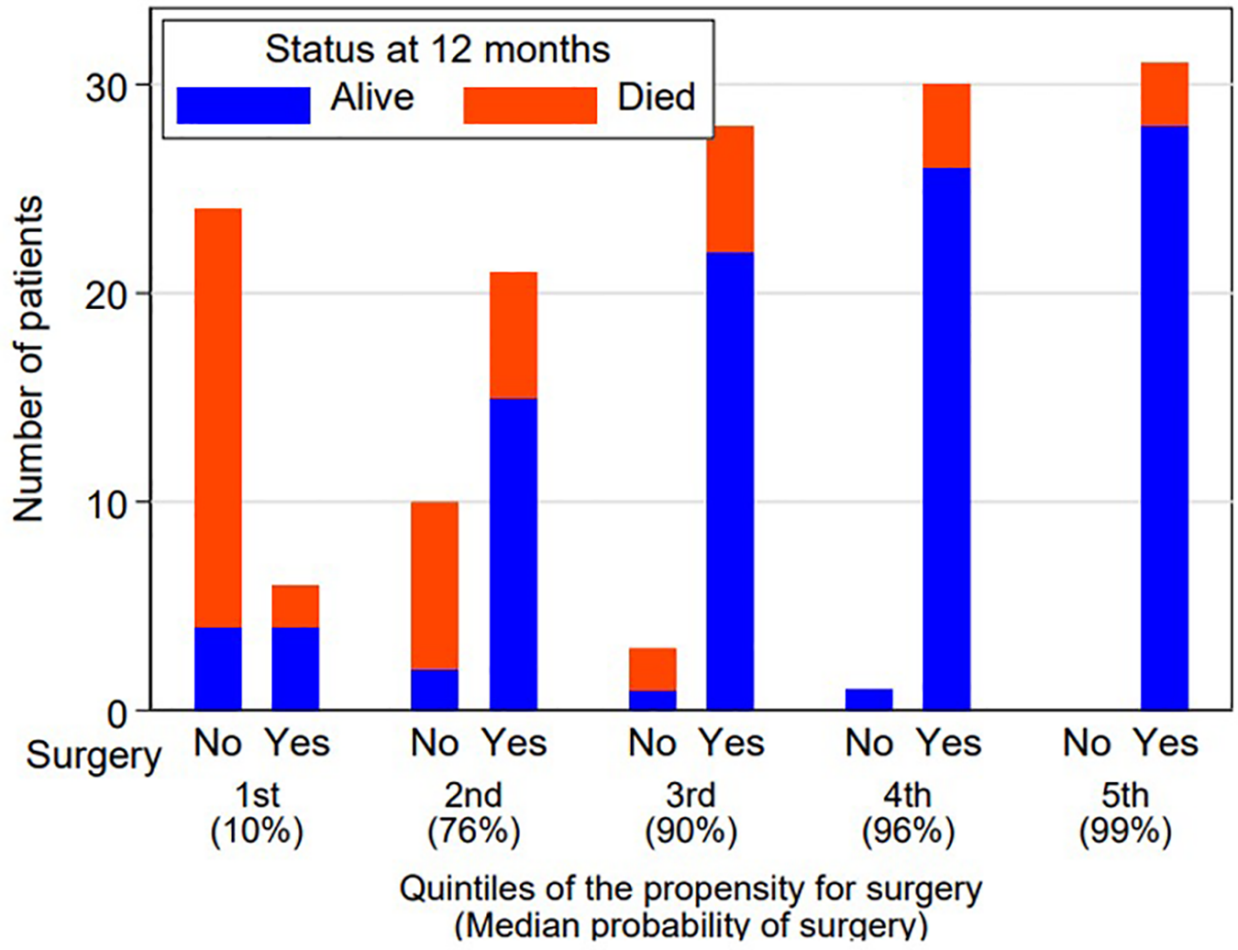
Survival by surgery and quintiles of propensity for surgery. Cox-model adjusted for the probability of surgery with propensity score.

We examined the positive effect of surgery on survival in a Cox model adjusted for the probability of surgery with the propensity score (in quintiles). Surgery continued to show a strong positive effect overall [HR 0.15 (95% CI 0.07–0.35)] and adjusted for quintiles (HR 0.20 [95% CI 0.09–0.43). Participants in the most favourable quintile had a median probability of surgery of 99% with a high rate of survival after one year (94%), whereas surgical candidates in the least favourable quintile had a median probability of surgery of 10% with a one-year mortality of 73% (Figure 4).

## Discussion

In this study, we analyzed 154 patients with ALSIE with a clear-cut operative indication according to Clinical Practice Guidelines. Patients that did not undergo surgery were older and had a higher risk score compared to those who received surgical treatment. Especially patients with dialysis and liver cirrhosis were often deemed unfit for surgery as carefully assessed by the Endocarditis Team. Non-operated patients had a worse prognosis with higher mortality rates up to one-year follow-up. The positive effect of surgery of survival remained after correcting for other risk factor, suggesting that guideline-based surgery represents a protective factor. However, the question arises whether surgical indication should also be pushed in those patients with a higher preoperative risk and therefore a higher expected perioperative mortality.

In the currently available literature, only few authors have studied outcomes of patients with ALSIE that fulfilled criteria for a surgical treatment but were finally treated conservatively (9–13). In the present series, this group represents 25% of the entire cohort and correlates to reports from others: Habib et al. (12) 26%, Iung et al. (13) 19%, Chu et al. (9) 24%, Ramos-Martínez et al. (11) 36%, and more recently Carino et al. (10) 26%. In an analysis of over 1,500 patients, Habib et al. (1) did not discriminate between left- and right-sided IE and determined worse outcomes with a 30-day mortality of 30% among not-operated patients (12). Other authors (9–11) studied specifically ALSIE patients and observed similar trends. Chu et al. (9) reported a 6-month mortality of 55%, Ramos-Martinez et al. (11) a 1-year mortality of 74.4%, and Carino et al. (10) noted 1- and 2-year mortality rates of 72% and 90%, respectively. Although mortality was high in these studies, there was no uniform follow-up. Only Ramos-Martinez et al. (11), and Carino et al. (10) reported information of or beyond 1 year. In our observation, mortality of patients who were candidates for surgery but finally received medical treatment only was 83% at 1 year. This is comparable to the aforementioned investigations.

In our study, preoperative characteristics of not-operated patients differed from those who underwent surgery: this population was older (62 vs. 70 years *p* < 0.001) and had more comorbidities, as reported by other authors (9–11). Furthermore, these patients more often presented initially in a worse clinical condition due to a higher rate of septic and cardiogenic shock and intubation rate. Similar findings were observed by Ramos-Martinez et al. (11) in a larger cohort. Even though heart failure, end-stage liver disease and septic shock have been found to be associated with poorer prognosis (and this not only in the setting of IE) (9, 11, 21, 22), decision to perform surgery in these cases remains challenging. Nevertheless some of these patients might benefit from surgery despite their poor preoperative condition.

Our multivariable logistic regression model showed that dialysis, liver cirrhosis and hemorrhagic stroke (trend) were more frequently found in the cohort of not-operated patients. This correlates to observations from Chu et al. (9). In these more complex scenarios, the importance of a multidisciplinary approach via the Endocarditis Team is extremely important when decision has to be taken on which approach should be followed to offer the most optimal treatment (1, 23). In this study, it seems that the Endocarditis Team more often declined surgery in patients perceived to have a short life expectancy as illustrated by the differences in baseline characteristics with a high CCI, EuroSCORE II and APORTEI in non-operated patients. Therefore, we assessed the difference in mortality by a propensity score and Cox regression analysis to compensate possible confounding factors by baseline differences.

Several score models are available to estimate the operative risk prior to surgery. Some have found that the use of EuroSCORE II might underestimate surgical mortality in the setting of infective endocarditis (24). Lately, Varela et al. (18) reported a new score for prognostic assessment in IE (25) which better estimated *de facto* survival compared to EuroSCORE I. Furthermore, a recent study showed better performance of the APORTEI score compared to EuroSCORE II (26). In our analysis, significant differences were found in EuroSCORE II (6 vs. 13%) and APORTEI (13 vs. 33%) with an even obvious difference in the latter. Notwithstanding, risk scores are just a part of the decision-making process and indication to proceed with surgery or medical treatment should not exclusively rely on a risk scoring system.

In terms of contraindications for surgery, liver cirrhosis has been reported to worsen prognosis (21). Patients with end stage liver-disease Child-Pugh B or C have worse prognosis than non-cirrhotic patients, whereas no difference regarding mortality was found between class Child-Pugh A vs. non-cirrhotic patients. Liver cirrhosis facilitates bacterial infection due to leucocyte dysfunction, phagocyte defects and blood stasis due to portal hypertension (27). Patients with cirrhosis are more prone to develop IE (21, 22) and the associated immune dysfunction (27) might explain worse outcomes. In our observation, 13 (34%) patients had more than one reason be excluded from surgery, suggesting that frequently the combination of several reasons might influence the decision whether to perform surgery or not. In 11 (30%) patients, high perioperative mortality was expected, and 9 (23%) patients did not undergo surgery mainly because to end-stage liver disease. The reasons to proceed with medical treatment and not with surgery may be multiple: Carino et al. (10) found advanced age (>84 years), end-stage liver disease and severe brain injury, whereas Chu et al. (9) recorded poor prognosis regardless of treatment, surgeons' preference not to perform surgery or stroke as the most common causes for surgical denial.

### Strengths and limitations

Strengths of our study are the prospective collection of all IE cases, risk factors and outcomes and the use of structured event reporting forms. This is a single-center, non-randomized, observational study with a limited number and heterogeneous group of patients. Further limitations are the imbalances both group (116 who underwent and 38 who received medical treatment only. This may have introduced bias. By assessing additional factors in bivariable models, none of these parameters seemed to introduce an unexpected shift in the point-estimates for the treatment effect of surgery. As this is the experience of a tertiary-care referral center, the population patterns and data may not be generalizable. There is no formal record linkage with other hospitals. Therefore, it cannot be completely excluded that clinical events (e.g., recurrent surgery, death) were not reported by participants. Outcome data are limited to one-year; therefore, we cannot make any statement about the long-term prognosis of patients.

## Conclusions

Patients with ALSIE that fulfilled criteria for an operative procedure but that ultimately received medical treatment are older, have more comorbidities and therefore a higher preoperative risk profile. One-year mortality in the non-operation cohort is high (83%). Surprisingly, ALSIE cause by staphylococcus was not a strong indication for surgery, as would be expected and the reasons for this are not completely clear. These findings reinforce the role of the Endocarditis Team in the decision-making process. Due to the strong positive effect of surgery on mortality, future studies assessing surgical candidates that finally did not undergo surgery are of utmost importance.

## Data Availability

The raw data supporting the conclusions of this article will be made available by the authors, without undue reservation.
